# R1205H (Vicenza) causes conformational changes in the von Willebrand factor D′D3 domains and enhances von Willebrand factor binding to clearance receptors LRP1 and SR-AI

**DOI:** 10.1016/j.jtha.2024.06.023

**Published:** 2024-07-10

**Authors:** Ferdows Atiq, Orla Rawley, Jamie M. O’Sullivan, Mehmet Özbil, Dearbhla Doherty, Niamh Cooke, Virginie Terraube, Alain Chion, Aamir Amin, Anne-Marije Hulshof, Bogdan Baci, Ciara Byrne, Hanan E. Aburawi, David Lillicrap, James S. O’Donnell

**Affiliations:** 1 Irish Centre for Vascular Biology, School of Pharmacy and Biomolecular Sciences, Royal College of Surgeons in Ireland, Dublin, Ireland; 2 Department of Pathology and Molecular Medicine, Queen’s University, Kingston, Ontario, Canada; 3 Computational Biochemistry Group, Gebze Technical University, Institute of Biotechnology, Gebze, Kocaeli, Turkey; 4 BioMedicine Design, Pfizer, Grange Castle, Dublin, Ireland; 5 National Coagulation Centre, St James’s Hospital, Dublin, Ireland

**Keywords:** enhanced VWF clearance, type 1C VWD, von Willebrand disease, von Willebrand factor, VWF-R1205H (Vicenza)

## Abstract

**Background:**

von Willebrand factor (VWF)-R1205H variant (Vicenza) results in markedly enhanced VWF clearance in humans that has been shown to be largely macrophage-mediated. However, the biological mechanisms underlying this enhanced clearance remain poorly understood.

**Objectives:**

This study aimed to investigate the roles of (i) specific VWF domains and (ii) different macrophage receptors in regulating enhanced VWF-R1205H clearance.

**Methods:**

*In vivo* clearance of full-length and truncated wild-type (WT)-VWF and VWF with R1205 substitutions was investigated in *VWF*^−/−^ mice. Plate-binding assays were employed to characterize VWF binding to purified scavenger receptor class A member 1 (SR-AI), low-density lipoprotein receptor–related protein-1 (LRP1) cluster II or cluster IV receptors, and macrophage galactose-type lectin.

**Results:**

In full-length VWF missing the A1 domain, introduction of R1205H led to significantly enhanced clearance in *VWF*^−/−^ mice compared with WT-VWF missing the A1 domain. Importantly, R1205H in a truncated VWF-D′D3 fragment also triggered increased clearance compared with WT-VWF-D′D3. Additional *in vivo* studies demonstrated that VWF-R1205K (which preserves the positive charge at 1205) exhibited normal clearance, whereas VWF-R1205E (which results in loss of the positive charge) caused significantly enhanced clearance, pinpointing the importance of the positive charge at VWF-R1205. *In vitro* plate-binding studies confirmed increased VWF-R1205H interaction with SR-AI compared with WT-VWF. Furthermore, significantly enhanced VWF-R1205H binding to LRP1 cluster IV (*P* < .001) and less marked enhanced binding to LRP1 cluster II (*P* = .034) was observed. In contrast, VWF-R1205H and WT-VWF demonstrated no difference in binding affinity to macrophage galactose-type lectin.

**Conclusion:**

Disruption of the positive charge at amino acid R1205 causes conformational changes in the VWF-D′D3 domains and triggers enhanced LRP1-mediated and SR-AI–mediated clearance.

## 1 | INTRODUCTION

Enhanced von Willebrand factor (VWF) clearance represents a common pathologic mechanism in patients with type 1 and type 2 von Willebrand disease (VWD) [1–4]. A variety of different *VWF* missense sequence variants have been associated with pathologic increased clearance [1,2,5–7]. The VWD-Vicenza variant (c.3614G>A) results in markedly enhanced VWF clearance in humans and is characterized by an arginine (R) to histidine (H) substitution at position 1205 in the E3 module of the VWF-D3 domain [5,6,8]. Patients with VWD-Vicenza typically have reduced plasma VWF antigen (VWF:Ag) levels of less than 10 IU/dL, together with significantly elevated VWF propeptide–to–VWF:Ag ratios [8–10]. Although plasma VWF:Ag levels increase in response to desmopressin in VWD-Vicenza individuals, the plasma half-life of secreted VWF-R1205H is markedly reduced compared with wild-type (WT)-VWF [8,10,11]. In addition, 2 other VWF-R1205 substitutions (with cysteine and serine, respectively) have also been reported in VWD patients with markedly enhanced VWF clearance following desmopressin treatment [12,13].

*In vivo* clearance studies have demonstrated that the VWF-R1205H substitution plays a direct role in triggering enhanced VWF clearance [12,14,15]. In particular, the half-life of VWF-R1205H in *VWF*^−/−^ mice was significantly reduced compared with WT-VWF [12,14,15]. Similarly, VWF-R1205C and VWF-R1205S were also associated with significantly increased *in vivo* clearance compared with WT-VWF [12]. Interestingly, another conserved R is located at residue 1204 in human VWF. However, in contrast to the markedly enhanced clearance phenotype observed with VWF-R1205H, clearance of VWF-R1204H in *VWF*^−/−^ mice was not significantly different compared with WT-VWF [12]. Collectively, these findings demonstrate that VWF-R1205 plays a specific role in regulating VWF clearance *in vivo*.

The molecular mechanisms through which R1205H in VWF-D3 domain results in pathologic enhanced VWF clearance have not been fully defined, but accumulating evidence points to a role for macrophages. Mice clearance studies involving recombinant human VWF demonstrated that the rapid clearance of VWF-R1205H is macrophage-mediated [12]. In addition, Wohner et al. [16] reported enhanced binding of VWF-R1205H to the macrophage scavenger receptor class A member 1 (SR-AI). In this study, we utilized a series of recombinant VWF variants and truncations to elucidate the mechanisms through which R1205H triggers enhanced macrophage-mediated VWF clearance *in vivo*. In particular, we examined (i) the role of specific VWF domains in modulating increased clearance and (ii) the contribution of different macrophage receptors in regulating enhanced VWF-R1205H clearance.

## 2 | MATERIALS AND METHODS

### 2.1 | VWF expression and purification

**FIGURE 1:**
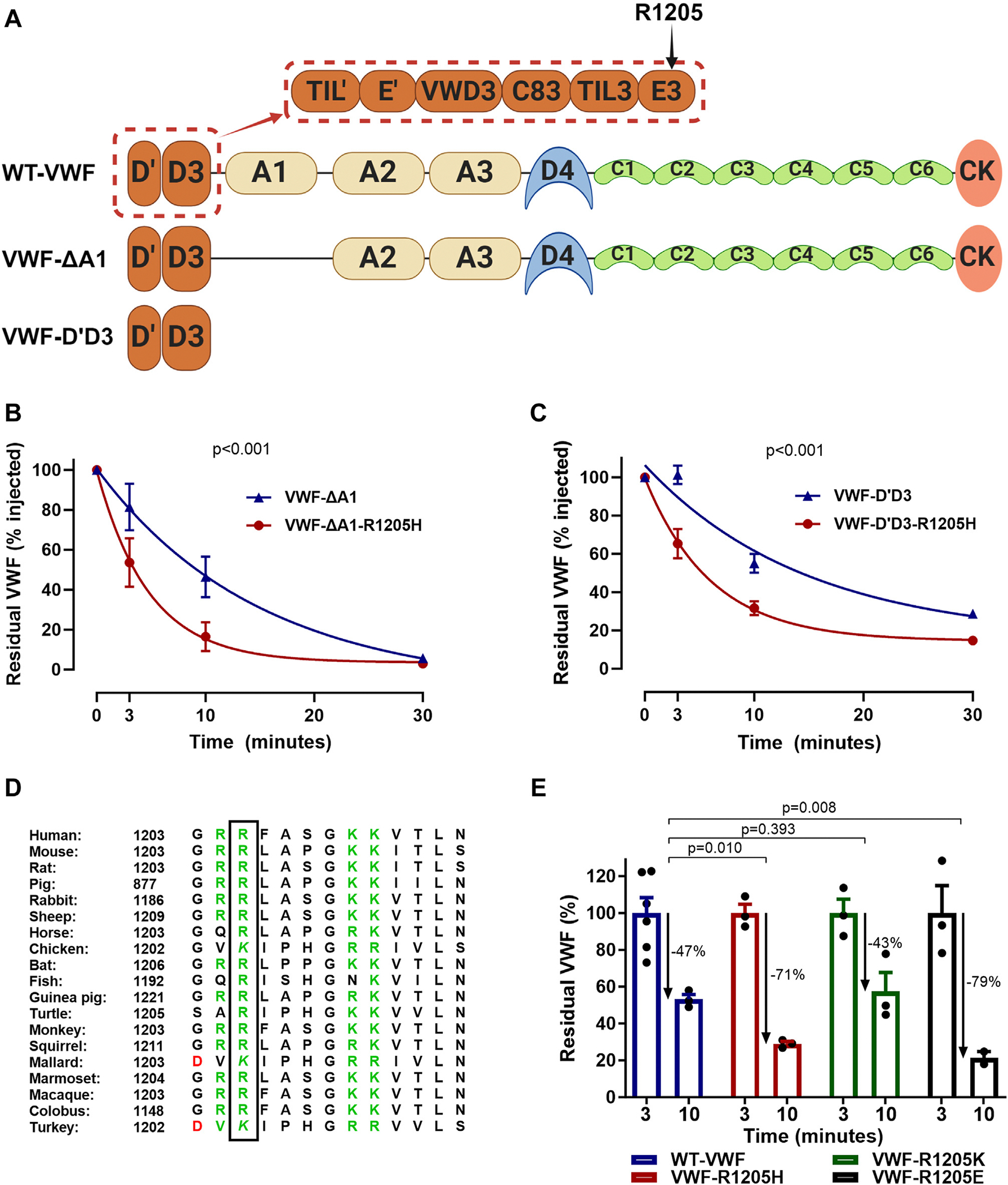
R1205 in the D3 domain regulates von Willebrand factor (VWF) clearance through local D′D3 effects. (A) Illustration of the domain structure of wild-type (WT)-VWF and the domains present in the truncated VWF fragments used in this study including VWF missing the A1 domain (VWF-ΔA1) and VWF-D′D3. In addition, the location of the R1205H substitution in the E3 domain of the D3 domain is highlighted. (B) VWF-ΔA1 and VWF-ΔA1-R1205H were expressed in human embryonic kidney (HEK)293T-antigen (T) cells. *In vivo* clearance was assessed in *VWF*^−/−^ mice following intravenous tail vein injection. Data are presented as mean ± SEM. *P* value is the outcome of the extra-sum-of-squares F test. (C) VWF-D′D3 and VWF-D′D3-R1205H were expressed in HEK293 freestyle (F) cells. *In vivo* clearance was assessed in *VWF*^−/−^ mice following intravenous tail vein injection. Data are presented as mean ± SEM. *P* value is the outcome of the extra-sum-of-squares F test. (D) Positive charge at VWF amino acid position 1205 is conserved in humans and other species through the presence of arginine or lysine. Positively charged residues are highlighted in green, and negatively charged residues are highlighted in red. (E) VWF-R1205K (with preserved positive charge) and VWF-R1205E (with loss of positive charge) were expressed in HEK293T cells. *In vivo* clearance was assessed in *VWF*^−/−^ mice following intravenous tail vein injection. Plasma samples were collected at 3 and 10 minutes after intravenous administration. The clearance rate of VWF from 3 to 10 minutes was assessed. R1205K, which has preserved positive charge, demonstrated similar clearance to WT-VWF, whereas R1205E, which has loss of positive charge (similar to R1205H), demonstrated significantly enhanced VWF clearance. *P* values are outcomes of Mann–Whitney U-test.

The previously published expression vector plasmid cloning DNA–VWF, containing the human *VWF*–complementary DNA, was utilized in all experiments [17]. Point mutations (R1205H, R1205K, R1205E, and R1204H) were generated in full-length VWF, VWF-D′D3, and A1 domain–deleted VWF (VWF-ΔA1) constructs (Figure 1A) using the KOD Hot Start DNA Polymerase (EMD Millipore). Full-length and truncated VWF constructs were transiently expressed in human embryonic kidney 293T cells using polyethylenimine as a transfection reagent. After 72 hours, conditioned serum-free medium was harvested, concentrated, and then purified utilizing nickel affinity chromatography, in accordance with established protocols [18]. TheVWF-D′D3 construct was provided by Pfizer and was transiently expressed in human embryonic kidney 239F cells using ExpiFectamine 293 (ThermoFisher Scientific) as a transfection agent. After 5 days, condition serum-free media was harvested and purified utilizing nickel affinity chromatography, followed by anion-exchange chromatography. Analytical size-exclusion chromatography was performed to remove any aggregates and ensure a homogenous monomeric population of protein.

### 2.2 | VWF clearance studies in *VWF*^−/−^ mice

Animal studies were approved by the Animal Research Ethics Committee of the Royal College of Surgeons in Ireland (REC1585) and were performed in accordance with the Irish Health Product Regulatory Authority (AE19127/P060). *VWF*^−/−^ mice on a C57Bl/6 background, initially obtained from the Jackson Laboratory, were used. *VWF*^−/−^ mice received intravenous injections of 30 nM VWF or VWF fragments diluted in 100 μL sterile-filtered phosphate-buffered saline. Mice were anesthetized with 2.5% tribromethanol (0.2 mL per 10 g of body weight) and exsanguinated by subclavicular incision at designated time intervals. Blood samples were collected into lithium heparin–coated microcontainers (BD Unitech), and VWF:Ag levels were quantified by enzyme-linked immunosorbent assay. The results of the clearance studies are presented as percentage residual VWF:Ag levels over time.

### 2.3 | *In silico* studies

X-ray crystal structure of monomeric human VWF-D′D3 assembly with a resolution of 2.50 Å (Protein Data Bank Identifier (PDB ID): 6N29) served as a starting 3-dimensional model [19]. This structure was stripped from its crystal water molecules and utilized for WT protein simulations. R1205H mutant was modeled from the same structure, substituting R 1205 residue with H on YASARA Structure software (Yasara Biosciences) [20,21]. Simulations were performed with GROMACS 5.1.4 software (KTH Royal Institute of Technology, Sweden), utilizing Chemistry at HARvard Macromolecular Mechanics version 27 (CHARMM27) forcefield [22,23]. Each protein was placed into a rectangular box with dimensions of 18.1 × 11.1 × 11.1 nm. These dimensions ensured that protein atoms stayed in the simulation box throughout the simulation. The box was filled with 4-point charge model (TIP4P) water molecules, and some of them were displaced during the addition of sodium and chloride ions to neutralize the system [24]. First, starting systems were subsequently energy-minimized using the steepest descent method for 50 000 steps. Then, energy-minimized structures were taken for the production phase. Molecular dynamics simulations without any constraints were carried out for 100 ns, with constant number of particles (N), pressure (P), and temperature (T), ie, NPT ensemble. The SETTLE algorithm was employed to constrain the bond length and bond angle of the solvent molecules, while the Linear Constraint Solver (LINCS) algorithm was used to constrain the bond length of the peptide [25,26]. Long-range electrostatic interactions were calculated by particle-mesh Ewald method [27]. A constant pressure of 1 bar was applied with a coupling constant of 1.0 ps, and water molecules/ions were coupled separately to a bath at 300 K with a coupling constant of 0.1 ps. The equation of motion was integrated at 2 fs time step using a leap-frog algorithm [28]. Production simulations were repeated twice for the total simulation time of 100 ns. The tools available in the GROMACS and VMD 1.9.1 software (The Theoretical and Computational Biophysics Group) were evaluated to analyze trajectories [29]. Clustering tool of GROMACS (gmx cluster) was used to obtain the most representative structures of 100 ns long trajectories. All the analyses, root mean square deviation, root mean square fluctuation, and radius of gyration (Rg), were performed by GROMACS tools.

### 2.4 | Macrophage receptor binding studies

Plate-binding studies were performed as previously detailed [16,30,31]. SR-AI and low-density lipoprotein receptor–related protein-1 (LRP1) cluster II were coated at 2 μg/mL, LRP1 cluster IV at 1 μg/mL and macrophage galactose-type lectin (MGL) at 5 μg/mL, as previously described [16,30,31]. VWF binding was probed using an horseradish peroxidase-conjugated rabbit polyclonal antibody (Dako), for which a 1:2000 dilution was used for LRP1 cluster IV and LRP1 cluster II and 1:1000 for SR-AI and MGL.

### 2.5 | Data presentation and statistical analysis

Data were plotted and analyzed with GraphPad Prism 10.1.0 (GraphPad Software LCC). Nonlinear regression curve fit was used to obtain clearance curves and plate-binding curves. Dots and error bars in clearance curves and binding curves depict mean values ± SEM. The extra-sum-of-squares F test was used to compare clearance rate of 2 clearance curves or to compare the binding capacity of 2 VWF constructs or VWF sequence variants. Mann–Whitney U-test was used to compare the VWF fall-off rate from 3 to 10 minutes for different VWF-R1205 substitutions. *P* values <.05 were considered statistically significant.

## 3 | RESULTS AND DISCUSSION

Previous studies demonstrated that the VWF-A1A2A3 truncation (Figure 1A) and full-length VWF were cleared at similar rates in *VWF*^−/−^ mice [14]. In contrast, the half-life of an extended VWF-D′D3A1A2A3 (VWF-D′A3) truncation (Figure 1A) was significantly prolonged compared with VWF-A1A2A3 [14]. These findings led to the hypothesis that the VWF-D′D3 domains may inhibit VWF-A1 domain–mediated clearance [14]. Importantly, we previously observed that introduction of R1205H into VWF-D′A3 resulted in significantly enhanced clearance [12]. To determine whether the R1205H substitution triggers a reduced half-life by abrogating an inhibitory effect of the D′D3 region on VWF-A1–mediated clearance, we first expressed recombinant VWF-ΔA1 with and without the R1205H substitution (Figure 1A). We observed that VWF-ΔA1-R1205H was cleared significantly faster (*P* < .001) in *VWF*^−/−^ mice compared with WT-VWF-ΔA1 (Figure 1B). Consequently, we next examined the effect of the R1205H substitution on *in vivo* clearance of a shorter VWF-D′D3 truncation (Figure 1A). Importantly, we observed that the half-life of VWF-D′D3-R1205H was significantly (*P* < .001) reduced compared with WT-VWF-D′D3 (Figure 1C). Collectively, these novel data demonstrate that the enhanced clearance phenotype associated with VWF-R1205H is modulated through local effects within the VWF-D′D3 region that are independent of the VWF-A1 domain.

Positively charged VWF-R1205 is highly conserved (Figure 1D) and surface exposed, leading to the possibility that it may be involved in charge-dependent interactions with other negatively charged amino acids within VWF. Consistent with the concept that charge at residue 1205 may be important, we observed that VWF-R1205 substitution with negatively charged glutamate (R1205E) resulted in significantly enhanced VWF fall-off from 3 to 10 minutes in *VWF*^−/−^ mice (Figure 1E). Conversely, conservation of the positive charge at position 1205 through the introduction of a lysine residue (VWF-R1205K) demonstrated a similar fall-off rate from 3 to 10 minutes as WT-VWF (Figure 1E). Together, these results highlight the important role of the positive charge at VWF amino acid 1205 in the clearance rate of VWF.

**FIGURE 2:**
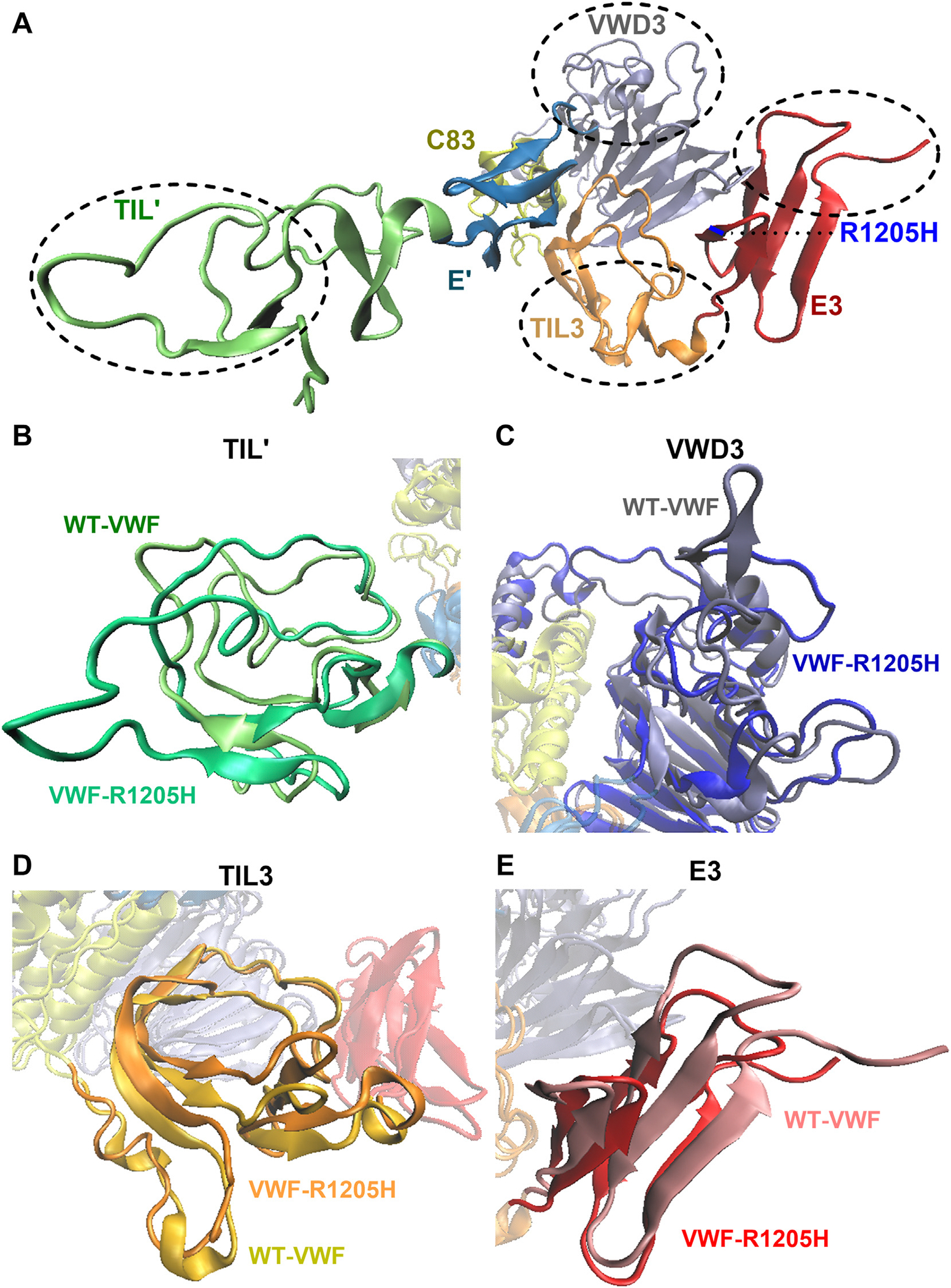
R1205H has significant effects on the confirmation of the von Willebrand factor (VWF)-D′D3 region. Average structures for wild-type (WT)-D′D3 and R1205H-D′D3 were aligned using molecular dynamics simulations. (A) Four distinct altered regions in VWF-R1205H were observed compared with WT-VWF. These regions include the TIL′ domain, the von Willebrand disease (VWD) 3 domain, the TIL3 domain, and the E3 domain, respectively, as encircled in the figure. (B) The conformation of the TIL′ domain is illustrated for R1205H (lime) and WT-VWF (light green). R1205H causes significantly altered conformation of the illustrated loops and β-sheet within the TIL′ domain compared with WT-VWF. (C) The conformation of the VWD3 domain is illustrated for R1205H (blue) and WT-VWF (dark gray). R1205H causes significantly altered conformation of the illustrated loops, α-helices, and β-sheets within the VWD3 domain compared with WT-VWF. (D) The conformation of the TIL3 domain is illustrated for R1205H (orange) and WT-VWF (yellow). R1205H causes significantly altered conformation of the illustrated α-helices and β-sheets within the TIL3 domain compared with WT-VWF. (E) The conformation of the E3 domain is illustrated for R1205H (red) and WT-VWF (salmon). R1205H causes significantly altered conformation of the illustrated loops and β-sheets within the E3 domain compared with WT-VWF.

To examine the effects of VWF-R1205H on VWF-D′D3 confirmation, classical molecular dynamics simulations were performed. The root mean square fluctuation analysis, which assesses the flexibility of the amino acid backbone, demonstrated significant reduction in the VWD3 and C83 subdomains (Supplementary Figure S1). Conversely, a slight increase in fluctuation was observed in the E3 subdomain, which contains the R1205H mutation site. Specifically, the region with increased flexibility included V1198 to F1206 and T1213 to I1224. Consistent with the *in vivo* findings, this increase in flexibility may be attributed to the loss of positive charge in VWF-R1205H. Next, Rg analysis was performed, revealing a slight increase in Rg in VWF-R1205H compared with WT-VWF (Supplementary Figure S2). This indicates that VWF-R1205H exhibits a less compact structure compared with WT-VWF. To understand the cause of this change, the average structures for WT-D′D3 and R1205H-D′D3 were aligned. Interestingly, 4 distinct altered regions in VWF-R1205H compared with WT-VWF were observed (Figure 2A). These included changes in the TIL′ domain, the D3 domain, the TIL3 domain, and the E3 domain, respectively (Figure 2B–E). Based on these findings, it is clear that the single R1205H substitution has significant effects on the conformation of the VWF-D′D3 region. Cumulatively, these findings suggest that the loss of the positively charged residue at amino acid 1205 in the E3 module of the VWF-D3 domain causes significant local changes in conformation that trigger pathologic enhanced clearance.

**FIGURE 3:**
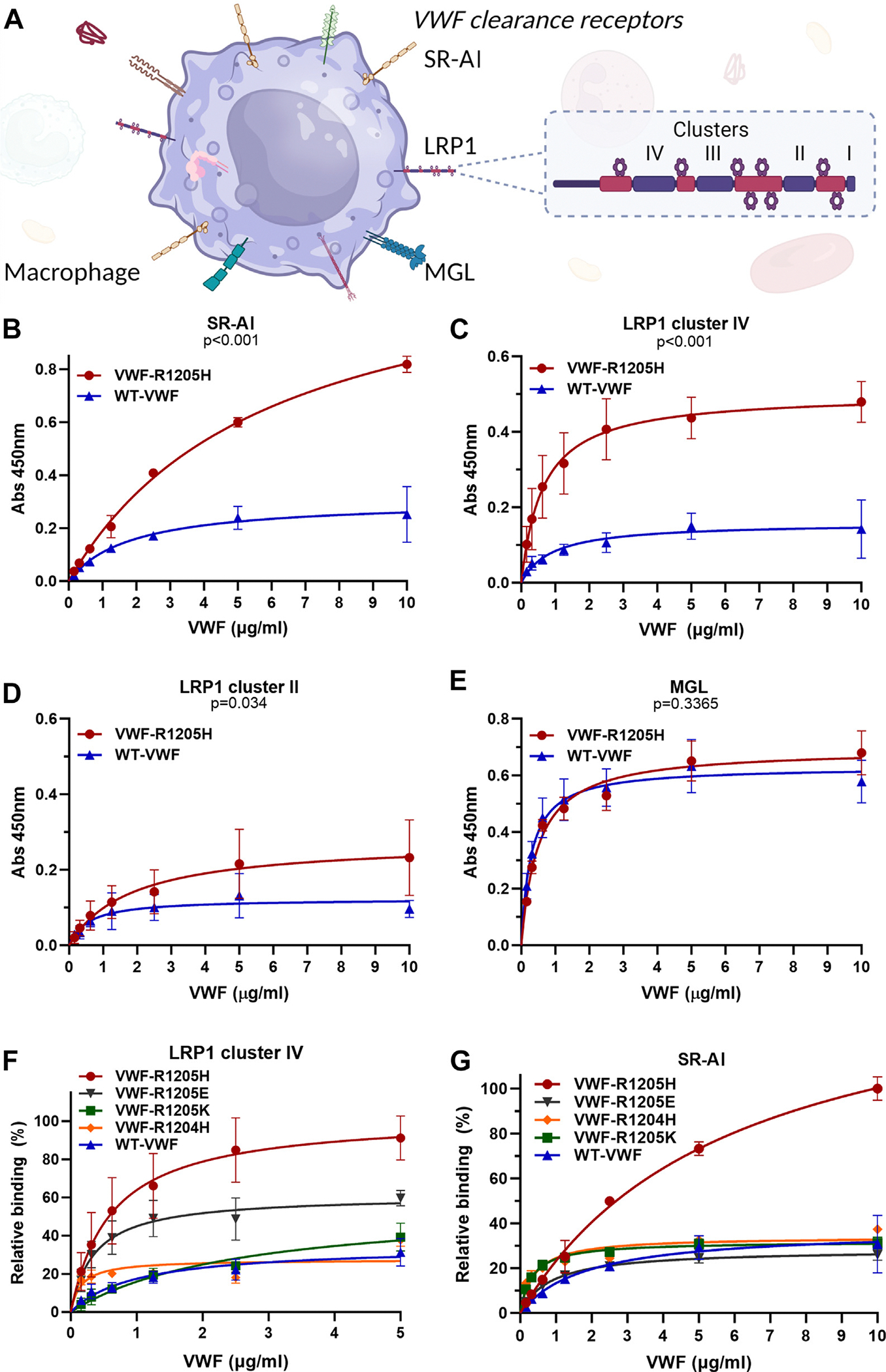
R1205 substitutions determine von Willebrand factor (VWF) interaction with macrophage scavenger receptor class A member 1 (SR-AI) and lipoprotein receptor–related protein-1 (LRP1) clearance receptors. (A) Several macrophage cellular receptors have been implicated in contributing to VWF clearance *in vivo*, including LRP1, SR-AI, and macrophage galactose-type lectin (MGL). (B–G) The effect of VWF-R1205 substitutions in regulating interaction with individual macrophage clearance receptors was assessed using immunosorbent plate-binding assays. (B) VWF-R1205H demonstrated significantly enhanced binding to SR-AI (2 μg/mL). compared with wild-type (WT)-VWF. Data are presented as mean absorbance (Abs) at 450 nm ± SEM. *P* value is the outcome of the extra-sum-of-squares F test. (C) VWF-R1205H demonstrated enhanced binding to LRP1 cluster IV (1 μg/mL) compared with WT-VWF. Data are presented as mean Abs at 450 nm ± SEM. *P* value is the outcome of the extra-sum-of-squares F test. (D) Although VWF binding to LRP1 cluster II (2 μg/mL) was less marked, nonetheless, VWF-R1205H binding was again significantly increased. Data are presented as mean Abs at 450 nm ± SEM. *P* value is the outcome of the extra-sum-of-squares F test. (E) No difference in binding of VWF-R1205H and WT-VWF to MGL (5 μg/mL) was observed. Data are presented as mean Abs at 450 nm ± SEM. *P* value is the outcome of the extra-sum-of-squares F test. (F) Loss of positive charge at residue 1205 (VWF-R1205H or VWF-R1205E) resulted in significantly enhanced binding to LRP1 cluster IV. Data are presented as mean normalized binding (%) ± SEM. *P* value is the outcome of the extra-sum-of-squares F test. If positive charge is conserved (VWF-R1205K), interaction with LRP1 cluster IV was similar to WT-VWF. (G) Although R1205H caused markedly increased SR-AI binding, no significant change in binding was observed for VWF-R1205K, -R1205E, or -R1204H compared with WT-VWF. Data are presented as mean normalized binding (%) ± SEM. *P* value is the outcome of the extra-sum-of-squares F test.

We previously showed that clodronate-induced macrophage depletion significantly prolonged the half-lives of VWF-R1205H, -R1205S, and -R1205C in *VWF*^−/−^ mice [12]. Several macrophage cellular receptors have been shown to interact with VWF, including LRP1, SR-AI, and MGL (Figure 3A) [16,32,33]. In keeping with previous reports, we observed that binding of VWF-R1205H to SR-AI in an immunosorbent plate-binding assay was markedly enhanced compared with WT-VWF (*P* < .001; Figure 3B). Previous studies have demonstrated that VWF can interact with extracellular cluster repeats II and cluster IV of the LRP1 scavenger receptor [30]. Interestingly, we observed that VWF-R1205H binding to LRP1 cluster IV was significantly (*P* < .001) increased compared with WT-VWF (Figure 3C). Although VWF binding to LRP1 cluster II was less pronounced, enhanced VWF-R1205H binding was again observed (*P* = .034; Figure 3D). It was recently shown that LRP1 is also present on liver sinusoidal endothelial cells in rats and may therefore potentially impact VWF clearance through cells other than macrophages [34,35]. Additional studies are needed to investigate whether LRP1 on liver sinusoidal endothelial cells affects VWF clearance in humans. In contrast to LRP1, binding of VWF-R1205H to the macrophage C-type lectin MGL was similar to that of WT-VWF (Figure 3E). This observation is consistent with the concept that O-linked glycans located on either side of the VWF-A1 domain are primarily responsible for regulating VWF interaction with MGL [31].

We next investigated how changes in charge at amino acid 1205 in the VWF-D3 domain influenced LRP1 and SR-AI interactions. In keeping with our *in vivo* clearance data, we observed that loss of positive charge (VWF-R1205H or VWF-R1205E) resulted in significantly enhanced binding to LRP1 cluster IV (*P* < .001; Figure 3F). In contrast, conservation of the positive charge (VWF-R1205K) resulted in a similar interaction with LRP1 cluster IV and SR-AI compared with WT-VWF (Figure 3F, G). Consistent with a key role for the specific VWF-R1205 residue in mediating enhanced clearance, VWF-R1204H binding to LRP1 and SR-AI was not significantly different compared with WT-VWF (Figure 3F, G). Interestingly, although VWF-R1205H caused markedly increased SR-AI binding, no significant change in binding was observed for VWF-R1205E compared with WT-VWF (Figure 3G). We hypothesize that different amino acid substitutions at VWF-R1205 may therefore have differing effects on VWF structure and consequently its interactions with clearance receptors. Consistently, previous studies reported that different VWF-R1205 substitutions (ie, H/S/C) and VWF-C1130 substitutions (ie, Y/F/G/R) were associated with significant differences in VWF clearance rates in human VWD patients [13,36].

In conclusion, our findings demonstrate that the single R1205H substitution in the E3 module of the VWF-D3 domain causes significant local conformational changes in the VWF-D′D3 region. These local changes subsequently trigger enhanced macrophage-mediated clearance that is modulated at least in large part through SR-AI and LRP1.

## Supplementary Material

Supplementary Material
