# Supplementary material for: R1205H (Vicenza) causes conformational changes in the von Willebrand factor D′D3 domains and enhances von Willebrand factor binding to clearance receptors LRP1 and SR-AI
Source: J Thromb Haemost. Author manuscript; Available in PMC 2025 Oct 1. (PMC11533894; doi:10.1016/j.jtha.2024.06.023)
Supplement: Supplementary Material [file NIHMS2027625-supplement-Supplementary_Material.docx]

R1205H (Vicenza) causes conformational changes in the VWF-D’D3 domains and enhances VWF binding to clearance receptors LRP1 and SR-A1.

Ferdows Atiq^1^*, Orla Rawley^2^*, Jamie M. O’Sullivan^1^*****, Mehmet Özbil^3^, Dearbhla Doherty^1^, Niamh Cooke^4^, Virginie Terraube^4^, Alain Chion^1^, Aamir Amin^1^, Anne-Marije Hulshof^1^, Bogdan Baci^1^, Ciara Byrne^1^, Hanan Aburawi^1^, David Lillicrap^2^^ and James S. O’Donnell^1,5^^

^1^ Irish Centre for Vascular Biology, School of Pharmacy and Biomolecular Sciences, Royal College of Surgeons in Ireland, Dublin, Ireland.

^2^ Department of Pathology and Molecular Medicine, Queen's University, Kingston, Canada.

^3^ Computational Biochemistry Group, Gebze Technical University, Institute of Biotechnology, 41400 Gebze, Kocaeli, Turkey.

^4^ BioMedicine Design, Pfizer, Grange Castle, Dublin, Ireland.

^5^ National Coagulation Centre, St James’s Hospital, Dublin, Ireland.

***/^ These authors contributed equally to this study.**

**SUPPLEMENTARY MATERIALS**

**Supplementary Figure S1. VWF-R1205H exhibits altered flexibility of the amino acid backbone at specific VWF residues compared to WT-VWF.**

**
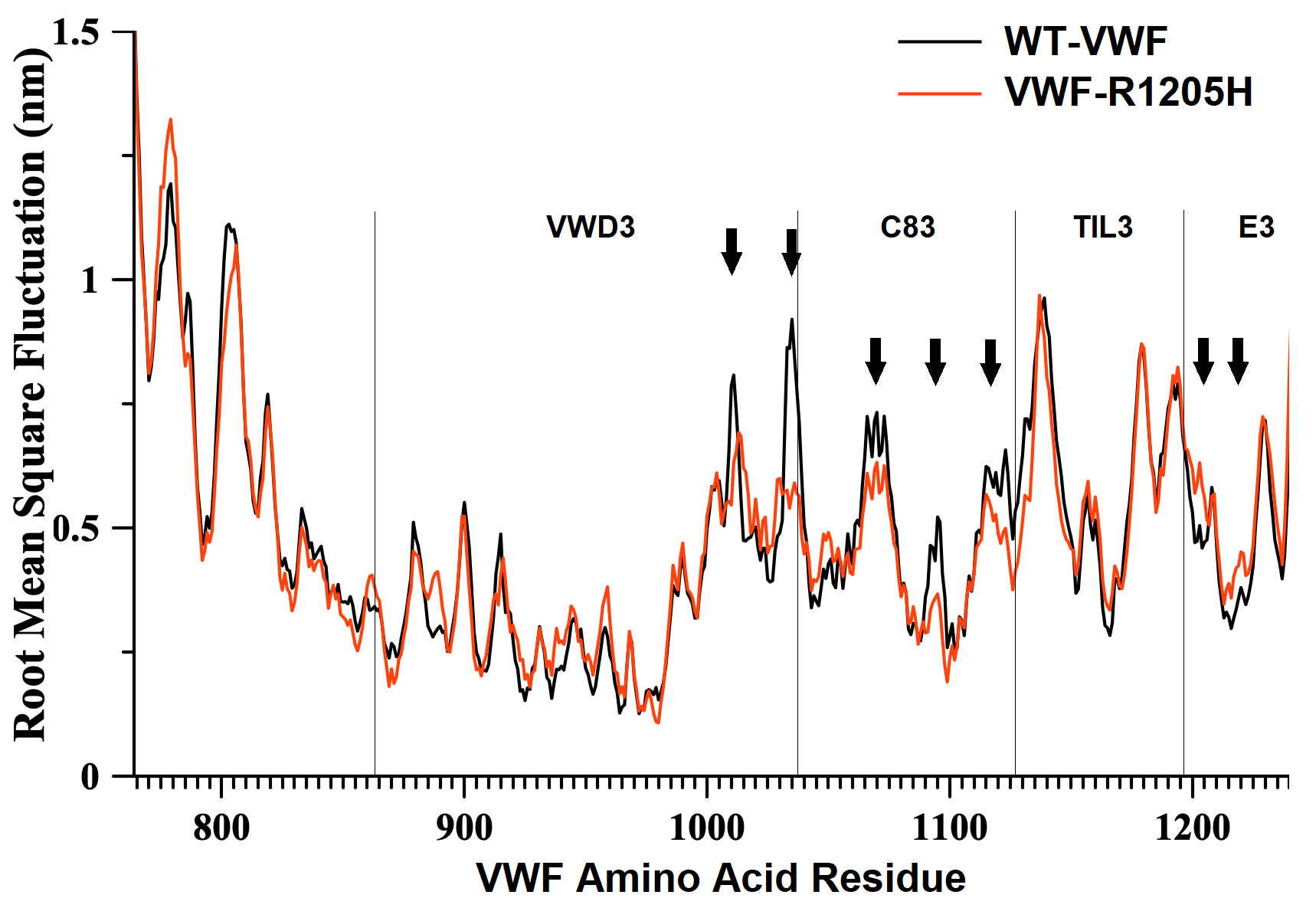
**

The flexibility of the amino acid backbone was assessed with root mean square fluctuation analysis utilizing GROMACS tools. The VWD3, C83, TIL3 and E3 subdomain amino acid boundaries are indicated with vertical lines. Arrows depict amino acid regions with significant altered root mean square fluctuation between VWF-R1205H and WT-VWF. VWF-R1205H demonstrated significant reduction in root mean square fluctuation in the VWD3 and C83 subdomains, whereas a slight increase in fluctuation was observed in the E3 subdomain.

**Supplementary Figure S2. Radius of gyration analysis was performed to assess the compactness of the structure of VWF-R1205H compared to WT-VWF.**

**
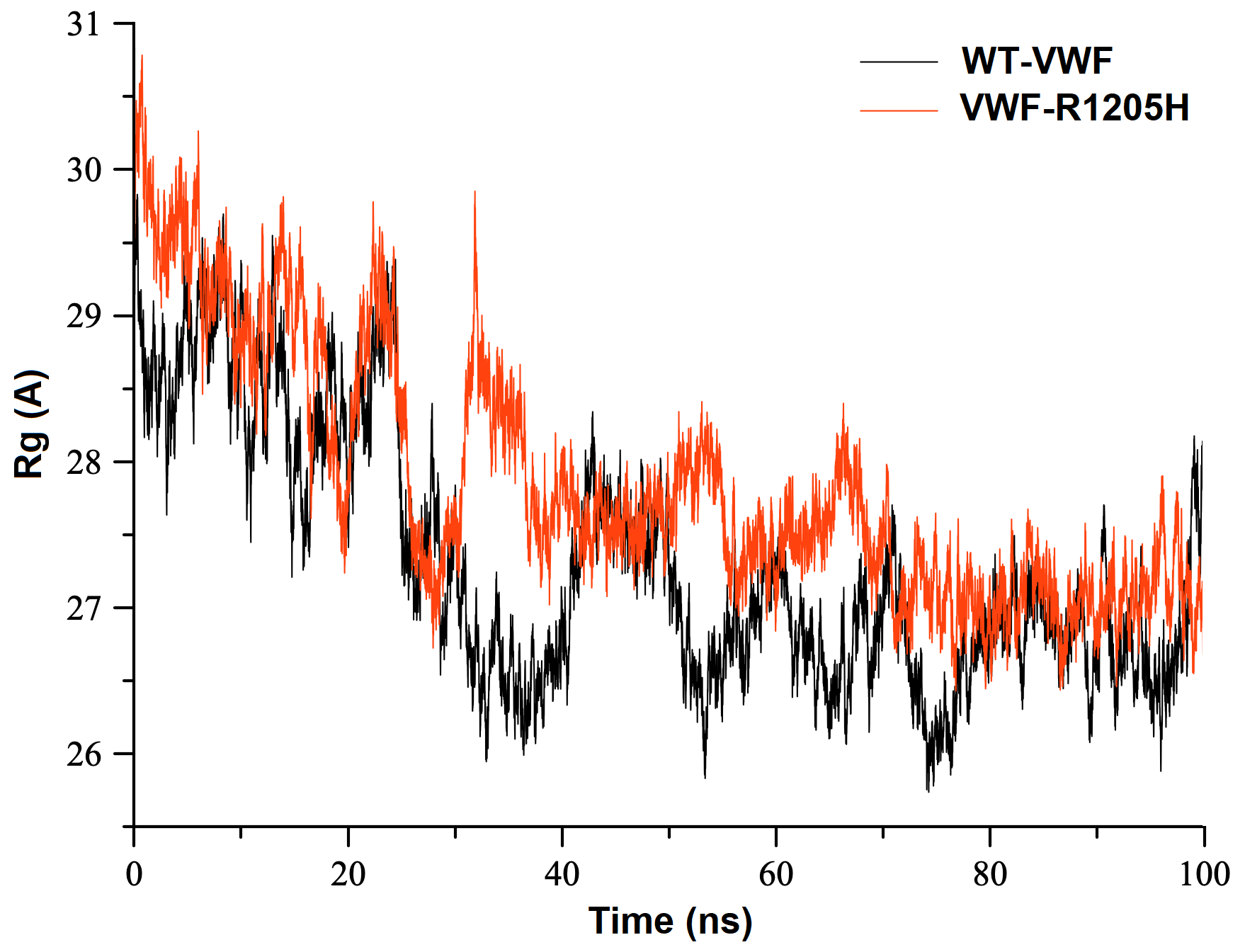
**

The compactness of the protein was assessed using radius of gyration analysis. GROMACS tools were employed to perform radius of gyration analysis. A slight increase in Rg was observed in VWF-R1205H compared to WT-VWF, indicating that VWF-R1205H exhibits a less compact structure compared to WT-VWF.
